# Infant feeding practices within a large electronic medical record database

**DOI:** 10.1186/s12884-017-1633-9

**Published:** 2018-01-02

**Authors:** Emily Bartsch, Alison L. Park, Jacqueline Young, Joel G. Ray, Karen Tu

**Affiliations:** 1 University of Toronto, Toronto, Canada; 2 Institute for Clinical Evaluative Sciences, Toronto, Canada; 3 Departments of Medicine, Health Policy Management and Evaluation, and Obstetrics and Gynecology St. Michael’s Hospital, University of Toronto, Toronto, Canada; 4 Department of Family and Community Medicine, and Health Policy Management and Evaluation, Toronto Western Hospital Family Health Team, University of Toronto, Toronto, Canada

**Keywords:** Breastfeeding, Formula feeding, Infant nutrition, Electronic medical record, EMR

## Abstract

**Background:**

The emerging adoption of the electronic medical record (EMR) in primary care enables clinicians and researchers to efficiently examine epidemiological trends in child health, including infant feeding practices.

**Methods:**

We completed a population-based retrospective cohort study of 8815 singleton infants born at term in Ontario, Canada, April 2002 to March 2013. Newborn records were linked to the Electronic Medical Record Administrative data Linked Database (EMRALD™), which uses patient-level information from participating family practice EMRs across Ontario. We assessed exclusive breastfeeding patterns using an automated electronic search algorithm, with manual review of EMRs when the latter was not possible. We examined the rate of breastfeeding at visits corresponding to 2, 4 and 6 months of age, as well as sociodemographic factors associated with exclusive breastfeeding.

**Results:**

Of the 8815 newborns, 1044 (11.8%) lacked breastfeeding information in their EMR. Rates of exclusive breastfeeding were 39.5% at 2 months, 32.4% at 4 months and 25.1% at 6 months. At age 6 months, exclusive breastfeeding rates were highest among mothers aged ≥40 vs. < 20 years (rate ratio [RR] 2.45, 95% confidence interval [CI] 1.62–3.68), urban vs. rural residence (RR 1.35, 95% CI 1.22–1.50), and highest vs. lowest income quintile (RR 1.18, 95% CI 1.02–1.36). Overall, immigrants had similar rates of exclusive breastfeeding as non-immigrants; yet, by age 6 months, among those residing in the lowest income quintile, immigrants were more likely to exclusively breastfeed than their non-immigrant counterparts (RR 1.43, 95% CI 1.12–1.83).

**Conclusions:**

We efficiently determined rates and factors associated with exclusive breastfeeding using data from a large EMR database.

**Electronic supplementary material:**

The online version of this article (10.1186/s12884-017-1633-9) contains supplementary material, which is available to authorized users.

## Background

Breastmilk is recognized as the optimal form of infant nutrition, and has been shown to confer significant benefit to both the mother and infant [1–3]. For instance, exclusive breastfeeding is protective against infection and gastroenteritis in infancy [2, 3], and may prevent the development of asthma [2] and childhood obesity [4] in the long-term. Despite the benefits, and consequent recommendations from the World Health Organization (WHO) [5] and the Canadian Paediatric Society [6], according to the 2009 Maternity Experiences Survey (MES), only 14% of Canadian infants are exclusively breastfed at 6 months [7]. The 2009 Canadian Community Health Survey (CCHS) reported rates of exclusive breastfeeding in the first 6 months ranging from 22% to 33% from 2011 to 2014 [8]. However, the MES used telephone interviews to gather information from a stratified random sample, while the CCHS employs a cross-sectional, voluntary questionnaire.

Preterm infants are less likely to be breastfed [9, 10]. Of note, infants of immigrant women to Canada – who comprise 35% of all births in Ontario [11] – may not have significantly different rates of breastfeeding than those of Canadian-born women [12]. Worldwide, the WHO reports that return to work is the largest barrier to breastfeeding [13].

The increasing use of electronic medical records (EMR) in primary care provides an opportunity to efficiently explore feeding practices in large general populations, perhaps with less bias than may occur in self-report surveys or registries.

We set out to determine rates of exclusive breastfeeding and sociodemographic factors that relate to exclusive breastfeeding of Ontario infants.

## Methods

We performed a retrospective population-based cohort study using administrative datasets linked using unique encoded identifiers and analyzed at the Institute for Clinical Evaluative Sciences (ICES). We considered singleton term infants born in an Ontario hospital between April 1, 2002 and March 31, 2013, whose mother was an Ontario resident at the time of the birth. Maternal-newborn pairs were identified from the ICES MOMBABY dataset, which links the inpatient records of delivering mothers and their newborns in the Canadian Institute for Health Information (CIHI) Discharge Abstract Database (DAD), housed at ICES. Our study cohort was further limited to infants who had at least 1 postnatal visit with a family physician within 190 days of age in the Electronic Medical Record Administrative data Linked Database (EMRALD™), also housed at ICES. The EMRALD™ dataset was created using data from participating family practice EMRs across Ontario [14]. Specific to our study, EMRALD™ contains data for well-baby visits to family physicians, including the date of each visit, the infant’s corresponding age, anthropometric measures, feeding practices, developmental milestones and physical exams. Multi-fetal pregnancies were excluded as they are more prone to indicate preterm birth, small-for-gestational age, and specialized pediatric postnatal care.

To obtain information about feeding practices, we formulated an algorithm combining free text searches and structured field searches from the Rourke Baby Record, which is described at http://www.rourkebabyrecord.ca/pdf/RBR%202017%20Ontario%20English%20-%20Black%20171004.pdf and http://rourkebabyrecord.ca/default.asp. The Rourke Baby Record is a standardized and commonly used method for family physicians in Canada to record well baby visits in newborn and infant medical records. The content of the search algorithm is listed in the Additional file 1. The electronic search algorithm was used to abstract information about mode of infant feeding from all EMRs with a Rourke Baby Record. To find Rourke Baby Records the search algorithm looked in the free text progress notes made by the family physician for mention of a Rourke form. The name of the form can vary, so as many variations were accounted for, such as “Rourke”, “Well Baby Visit”, “Well Baby Check Up”, “Newborn Visit”, “1 month visit”, etc. If a Rourke Baby Record was found, the algorithm to search for documentation of feeding was then applied. Those EMRs without a Rourke Baby Record were manually abstracted for similar details about infant feeding. We included records up to 750 days of age in order to capture historical information on type and duration of feeding recorded in non-Rourke entries, as well as Rourke Baby Records with exclusive breastfeeding documented beyond 6 months, from which we inferred exclusive breastfeeding at earlier time points.

Three trained abstractors performed the manual EMR abstraction. The initial charts were also reviewed by a content expert (ALP) to correct for any inconsistencies. Both intra and inter-rater reliability were assessed for 5 % of the charts.

Typically, well baby visits to primary care providers occur shortly after birth and at 2 months, 4 months and 6 months, according to the Ontario infant immunization schedule [15]. As such, we chose these time points to assess the rates of exclusive breastfeeding. The denominators for the rates at 2, 4 and 6 months included infants with any visit at ≥ 60 days, ≥ 122 days, and ≥ 182 days of age, respectively. We chose the visit closest to but not preceding the target age for each time point. For children who did not have visits at all three time points, we estimated feeding status based on that documented at future visits. For example, if a child had a visit at 60 days then their 2 month feeding status was determined from that visit. If, however, their next visit was not until 182 days or more, then we determined if they were currently exclusively breastfeeding (from a Rourke record) or were previously exclusively breastfeeding (from a progress note) and we inferred that they were exclusively breastfeeding at 4 and 6 months.

To assess possible misclassification of exclusive breastfeeding as a result of inferring feeding status from future visits, we performed a complete case analysis of infants who had documented feeding at all three time points. Exclusive breastfeeding duration was calculated based on the date of birth and the date of the latest visit with confirmed exclusive breastfeeding.

Newborns to immigrant mothers were determined by linkage to the federal Immigration, Refugees and Citizenship Canada Permanent Resident Database held at ICES, which has records for permanent residents who immigrated to Ontario from 1985 to 2012. Neighborhood income quintile (Q) was determined by residential postal code at the time of birth, derived from Statistics Canada census data. Rurality was determined by the Registered Persons Database (RPDB) and maternal age at birth, parity and birthweight were determined by linkage with the MOMBABY database.

Rate ratios (RR) and 95% confidence intervals (CI) were calculated for each characteristic, comparing each group to that with the lowest rate (the reference group).

Statistical analysis was performed using SAS for UNIX, Version 9.4 (SAS Institute, Cary, NC), and EMR data analysis was performed in SQL Server 2012. The study was approved by the Sunnybrook Health Sciences Centre Research Ethics Board.

## Results

**Fig. 1:**
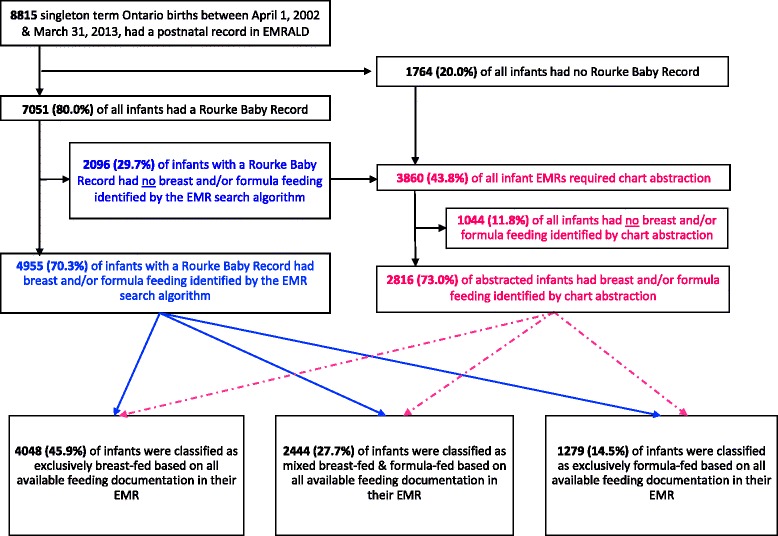
Derivation of the infant feeding cohort and validation cohort for the electronic medical record (EMR) search algorithm

We identified 8815 singleton term newborns with a birth record in MOMBABY and at least 1 postnatal visit in EMRALD™ before 190 days. The total number of visits for these infants was 110,794. In our study, 25,230 visits were included from infants with a Rourke Baby Record, and 54,856 visits were abstracted from the chart. The infants and mothers in EMRALD™ were similar to those across Ontario in terms of characteristics such as age and parity, however mothers in EMRALD™ were somewhat more likely to be a rural resident and Canadian-born, and less likely to be South or East Asian born and in lower income quintiles (Table 1). Of the 8815 infants in EMRALD™, 7051 (80.0%) had any Rourke Baby Record, while 1764 (20.0%) did not (Fig. 1). Among infants with a Rourke Baby Record, we identified infant feeding using the automated search algorithm for 4955 (70.3%) of patients. For the remainder of infants, manual abstraction was performed, wherein we identified feeding for 2816 additional infants. Feeding remained undetermined for 1044 (11.8%) of infants.

**Table 1:** Characteristics of all liveborn singleton infants and their mothers, born in an Ontario hospital between April 1, 2002 and March 31, 2013. Shown are births in the MOMBABY dataset vs. those concomitantly available in the EMRALD™ database

Characteristic	MOMBABY dataset (*N* = 1,204,042)	EMRALD™ dataset (*N* = 8,815)	Standardized difference
Mean (SD) age, years	29.9 (5.5)	30.2 (5.3)	−0.1
Median (IQR) parity	1 (1)	1 (1)	0.0
Rural residence, *n* (%)	125,850 (10.5)	2151 (24.4)	−0.4
Mother’s World region of birth, *n* (%)			
*Canada*	901,188 (74.9)	7719 (87.6)	−0.3
*Europe/Western*	47,586 (4.0)	358 (4.1)	0.0
*African/Caribbean*	38,508 (3.2)	132 (1.5)	0.1
*Middle East/N Africa*	30,679 (2.6)	120 (1.4)	0.1
*Latin America*	23,076 (1.9)	121 (1.4)	0.0
*South Asia*	94,458 (7.9)	133 (1.5)	0.3
*East Asia*	68,467 (5.7)	227 (2.6)	0.2
*Unknown*	80 (< 0.1)	≤ 5 (< 0.1)	0.0
Income quintile (Q), *n* (%)			
*Q1*	265,136 (22.0)	1540 (17.5)	0.1
*Q2*	240,322 (20.0)	1660 (18.8)	0.0
*Q3*	244,706 (20.3)	1922 (21.8)	0.0
*Q4*	249,328 (20.7)	1900 (21.6)	0.0
*Q5*	199,110 (16.5)	1756 (19.9)	−0.1
*Unknown*	5440 (0.5)	37 (0.4)	0.0
Mean (SD) birthweight, g	3461 (472)	3490 (464)	−0.1
Mean (SD) gestational age, weeks	39.2 (1.1)	39.3 (1.1)	−0.1
Infant male sex, *n* (%)	610,661 (50.7)	4527 (51.4)	0.0

The Cohen’s kappa statistic for inter-rater reliability of infant feeding classification was high (0.936). Based on all the automated and manually abstracted feeding results, 45.9% of infants were classified as exclusively breastfed, 14.5% exclusively formula-fed, and 27.7% mixed breastfed and formula-fed. The rates of breastfeeding were higher among infants in the automatically abstracted group than those of the manually abstracted group at 2, 4, and 6 months.

Rates of exclusive breastfeeding were 39.5% at 2 months, 32.4% at 4 months, and 25.1% at 6 months (Table 2). In a complete case analysis of 3959 infants who had visits at all three time points, the rates of exclusive breastfeeding did not significantly differ (data not shown). Breastfeeding rates were higher with greater maternal age, such that women over 30 were more than twice as likely as women under 20 to exclusively breastfeed at any time. Exclusive breastfeeding was more prevalent among urban than rural residents, women living in higher income neighbourhoods, and those who delivered in 2008 onward. Exclusive breastfeeding was not associated with parity, birthweight, or immigrant status; however, immigrant women living in the lowest income areas had significantly higher rates of breastfeeding than non-immigrant women living in the lowest income neighbourhood (Table 2).

**Table 2:** Rate and rate ratios for exclusive breastfeeding at 2 months, 4 months and 6 months, determined among infants in EMRALD™ who had documented feeding status

Characteristic	Age 2 months			Age 4 months			Age 6 months		
	No. of mother-infant pairs	Rate (%) of exclusive breastfeeding	Rate Ratio (95% CI)	No. of mother-infant pairs	Rate (%) of exclusive breastfeeding	Rate Ratio (95% CI)	No. of mother-infant pairs	Rate (%) of exclusive breastfeeding	Rate Ratio (95% CI)
Overall	7621	39.5	–	7416	32.4	–	7186	25.1	–
Age at delivery, years									
*< 20*	207	20.8	1.00 (referent)	201	15.4	Referent (1.00)	195	12.8	Referent (1.00)
*20–24*	979	28.7	1.38 (1.04–1.84)	950	20.8	1.35 (0.96–1.91)	924	16.2	1.27 (0.85–1.88)
*25–29*	2114	37.4	1.80 (1.37–2.36)	2054	29.9	1.94 (1.39–2.70)	1983	22.3	1.74 (1.20–2.54)
*30–34*	2691	43.7	2.10 (1.61–2.75)	2637	36.6	2.37 (1.71–3.29)	2557	28.5	2.23 (1.54–3.23)
*35–39*	1362	44.6	2.15 (1.63–2.82)	1315	37.6	2.44 (1.75–3.39)	1275	29.3	2.29 (1.57–3.33)
*≥ 40*	268	41.8	2.01 (1.49–2.72)	259	38.6	2.50 (1.75–3.58)	252	31.3	2.45 (1.62–3.68)
Parity									
*Multiparous*	4029	39.3	1.00 (referent)	3912	31.9	1.00 (referent)	3791	24.5	1.00 (referent)
*Primiparous*	3592	39.6	1.01 (0.95–1.07)	3504	32.9	1.03 (0.97–1.10)	3395	25.7	1.05 (0.97–1.14)
Urban residence									
*No*	1828	33.3	1.00 (referent)	1780	26.7	1.00 (referent)	1719	19.8	1.00 (referent)
*Yes*	5790	41.4	1.24 (1.16–1.34)	5633	34.2	1.28 (1.17–1.39)	5464	26.7	1.35 (1.22–1.50)
Immigrant									
*No*	6694	39.2	Referent	6514	32.1	1.00 (referent)	6309	24.7	1.00 (referent)
*Yes*	927	41.4	1.06 (0.97–1.15)	902	34.6	1.08 (0.98–1.19)	877	27.4	1.11 (0.98–1.24)
Residential income quintile (Q)									
*Q1 (lowest)*	1314	35.9	1.00 (referent)	1268	28.2	1.00 (referent)	1229	20.5	1.00 (referent)
*Q2*	1430	39.0	1.08 (0.98–1.19)	1386	32.5	1.15 (1.03–1.30)	1342	26.5	1.29 (1.12–1.49)
*Q3*	1706	39.5	1.10 (1.00–1.21)	1667	32.9	1.17 (1.04–1.31)	1617	26.1	1.27 (1.11–1.46)
*Q4*	1646	42.6	1.19 (1.08–1.30)	1608	35.9	1.27 (1.14–1.42)	1556	27.5	1.34 (1.17–1.54)
*Q5 (highest)*	1497	39.9	1.11 (1.01–1.22)	1461	31.8	1.13 (1.01–1.27)	1417	24.1	1.18 (1.02–1.36)
*Unknown*	28	21.4	0.60 (0.29–1.22)	26	15.4	0.55 (0.22–1.35)	25	8.0	0.39 (0.10–1.48)
Joint income Q/immigrant status									
*Q1 & non-immigrant*	1063	34.2	1.00 (referent)	1019	26.4	1.00 (referent)	985	18.9	1.00 (referent)
*Q1 & immigrant*	251	43.0	1.26 (1.07–1.48)	249	35.3	1.34 (1.10–1.63)	244	27.0	1.43 (1.12–1.83)
*Q2-Q5 & non-immigrant*	5613	40.2	1.17 (1.07–1.28)	5478	33.2	1.26 (1.13–1.40)	5307	25.9	1.37 (1.20–1.57)
*Q2-Q5 & immigrant*	666	41.0	1.20 (1.06–1.35)	644	34.6	1.31 (1.13–1.52)	625	27.7	1.47 (1.22–1.76)
*Unknown*	28	21.4	0.63 (0.31–1.28)	26	15.4	0.58 (0.24–1.44)	25	8.0	0.42 (0.11–1.61)
Birthweight, grams									
*< 2500*	92	34.8	1.00 (referent)	89	22.5	1.00 (referent)	83	19.3	1.00 (referent)
*2500 - < 3000*	895	39.7	1.14 (0.85–1.53)	858	33.1	1.47 (0.99–2.19)	830	27.1	1.41 (0.89–2.21)
*3000 - < 3500*	2872	39.0	1.12 (0.85–1.49)	2795	32.5	1.45 (0.98–2.13)	2702	25.1	1.30 (0.84–2.03)
*≥ 3500*	3762	39.9	1.15 (0.86–1.52)	3674	32.4	1.44 (0.98–2.12)	3571	24.7	1.28 (0.82–2.00)
Birth year									
*2002–2007*	1739	32.7	1.00 (referent)	1724	26.3	1.00 (referent)	1709	20.4	1.00 (referent)
*2008–2013*	5882	41.5	1.27 (1.18–1.36)	5692	34.2	1.30 (1.19–1.42)	5477	26.5	1.30 (1.17–1.45)

## Discussion

We examined exclusive breastfeeding rates among term singleton infants within a primary care EMR database. Previously, results from the MES, which used telephone administered questions, reported that 90% of women in Ontario intended to initiate breastfeeding, and 90% did so [16]. At 6 months, approximately half of women reported continuation of some form of breastfeeding, while under 15% were exclusively breastfeeding [7]. Using a questionnaire survey method, the CCHS found that approximately 27% of Ontario women reported exclusively breastfeeding at 6 months [8]. Like the CCHS, our current study found that the rate of exclusive breastfeeding at 6 months was 25%. Our results may be more representative of the general population, as they were taken from family medicine records of patients distributed throughout Ontario. Furthermore, by evaluating rates of breastfeeding over several time points, we observed a decrease in rates over time, especially between 4 and 6 months of age. At 2 months, 40% of infants were exclusively breastfed – a marked decrease from the 90% who initiate breastfeeding at birth [16].

As the uptake of EMRs continues to increase among primary care providers, data becomes more easily accessible, and the process of characterizing large cohorts becomes more efficient. Our study represents a large cohort of nearly 9000 infants – the largest study to date on breastfeeding in Ontario. The search algorithm we developed combined lists of free text terms and analysis of structured Rourke Baby Record fields. Although the search algorithm located feeding information for over half of the infants, over 40% further required manual chart abstraction, which is costly and time consuming. However the data developed here may be utilized for future studies that incorporate more sophisticated text mining and machine learning methods in order to allow for automated determination of breastfeeding rates in an automated time- and cost-efficient fashion. This will be beneficial for assessing changes in feeding practices over time, and the impact of breastfeeding promotion initiatives.

A few limitations herein need be acknowledged. Even after completing the manual chart abstraction, no feeding information could be obtained for over 1000 records. This suggests that there are inconsistencies in the way that EMRs, and standardized forms like the Rourke Baby Record, are completed. This highlights some challenges in using EMR data for secondary purposes. A second limitation of using EMR data was the resultant discrepancy in rates of breastfeeding between the infants in the automatic abstraction group compared to the manual abstraction group; however, the higher rate of breastfeeding with the automated algorithm was expected, given that all charts were put through an automated data extraction as an initial pass. Third, although the patients in EMRALD™ appeared to be ethnically diverse, mothers from South Asia and East Asia were under-represented, and rural dwelling women were over-represented in EMRALD™, compared to the entire population of Ontarians. Despite its differences from the Ontario population, EMRALD™ may be the most closely representative dataset available, as the data do not come from a specialty practice, and comprises multiple clinics and physicians across Ontario. Last, we were unable to assess the impact of maternal education or employment on exclusive breastfeeding rates, as this information was not available in our data.

## Conclusions

Among infants registered within a large primary care EMR, the rate of exclusive breastfeeding declined from 40% to 25% between two and 6 months of age. Exclusive breastfeeding was more likely among mothers who were older and residing in an urban and higher income quintile neighbourhood, and among immigrant women living in a lower income quintile neighborhoods. Our study suggests that primary care EMR data can be used to assess breastfeeding practices in large populations.

## Additional file

Additional file 1: Terminology used to identify feeding status in an automated fashion. Broadly, the search query contained distinct terms related to breastfeeding, formula feeding, and the Rourke Baby Record. Each of these categories was then subdivided with the terms below comprising the final algorithm to classify newborn feeding status. (DOCX 101 kb)

## Abbreviations

CCHS: Canadian community health survey
CIHI: Canadian institute for health information
DAD: Discharge abstract database
EMR: Electronic medical record
EMRALD™: Electronic medical record administrative data linked database
ICES: Institute for clinical evaluative sciences
MES: Maternity experiences survey
MOHLTC: Ministry of health and long-term care
RPDB: Registered persons database
WHO: World health organization
