# Supplementary material for: Infant feeding practices within a large electronic medical record database
Source: BMC Pregnancy Childbirth. 2018 Jan 2;18:1. doi: 10.1186/s12884-017-1633-9 (PMC5749017; doi:10.1186/s12884-017-1633-9)
Supplement: Additional file 1: — Terminology used to identify feeding status in an automated fashion. Broadly, the search query contained distinct terms related to breastfeeding, formula feeding, and the Rourke Baby Record. Each of these categories was then subdivided with the terms below comprising the final algorithm to classify newborn feeding status. (DOCX 101 kb) [file 12884_2017_1633_MOESM1_ESM.docx]

**Supplemental file 1.** Terminology used to identify feeding status in an automated fashion. Broadly, the search query contained distinct terms related to breastfeeding, formula feeding, and the Rourke Baby Record. Each of these categories was then subdivided with the terms below comprising the final algorithm to classify newborn feeding status.

| **Type of infant feeding** | **Included terminology** | **Excluded terminology** |
| --- | --- | --- |
| *Breast* | *Free text search words:*  breastfeed, breast feed, breast fed, lactat%, breastmilk, breast milk, breast feeding on demand, breast feedings on demand, continue breast feeding, breast feeding x [1,2,3,4,5,6,7,8,9,0], breastmilk with bottle, pumping and bottling, breast milk (pump%, pump and bottle, breast, yes, once a day, pump%, nurs%, done nurs%, very little, on demand, bid, qhs, q[1,2,3,4,5,6,7,8,9], [1,2,3,4,5,6,7,8,9,0] oz, full time, nurs*, pump*, wean*, exclusively, expressed milk  *Structured Rourke Baby Record fields:*  diet, Nutrition – breast feeding-vit D 10mcg/400IU/d, breast feeding, Nutrition – breast feeding and vitamin D, Nutrition – breast feeding(#/d), Nutrition – breast feeding: (Vit D), Breast feeding (Vit D 400IU/day), Breast feeding (Vit D 10 mcg/400 IU/d), Breast feeding (Vit D 10mcg/400 IU/d) | *Free text search words:* breast feeding clinic, Nutrition – breast feeding:no |
| *Formula* | *Free text search words:*  bottle feed, bottle fed, formula milk, formula fed, bottle formula [1,2,3,4,5,6,7,8,9,0], enfa%, enfamil%, enfapro%, ok, y, similac, PC with iron, lactose free formula, G/S, isomil, soy, goodstart, good start, walmart, wal%mart, parent%choice, parents choice, nestle, PC formula, [1,2,3,4,5,6,7,8,9,0], supplement*, president’s choice, nutramigen, exclusively, ###cc/q 3 hrs, ##-##cc per feed  *Structured Rourke Baby Record fields:*  Formula Feeding (Iron fortified):, Formula Feeding (Iron fortified) -, Feeding:, Formula Feeding (Iron fortified; 150ml=5oz/kg/day) |  |
| *Either breast or formula* | *Rourke Baby Record search words:*  Rourk%, Rour%, Rou%, Well Baby Vis%, Well B%, Newborn Baby Visit, WBC, Newborn – Follow Up, Well Baby Check Up, Well Baby Checkup, W0[0,1,2,3,4,5,6,7,8,9], New B0[1,2,3,4,5,6,7,8,9], 12-13 Mos%, [1,2,4,6,8,12,15,18] month visit;%, [1,2,4,6,8,12,15,18] month visit -%, [1,2,4,6,8,12,15,18] month visit;%, [1,2,4,6,8,12,15,18] months;%, [1,2,4,6,8,12,15,18] months;%, [1,2,3,4,5,6,7,8,9,12,15,18,19]%m%WBV[-,;]%,[1,2,3,4,5,6,7,8,9,12,15,18,19]-m%WBV[-,;]% |  |
